# Next generation technologies to improve post crash response time and quality

**Published:** 2019-08

**Authors:** Zeynep Sofuoglu, Turhan Sofuoglu

**Affiliations:** ^*a*^Izmir Democracy University, School of Medicine, Department of Public Health, Turkey.; ^*b*^Emergency Ambulance Physicians Assosciation, Turkey.

## Abstract

The number of mobile phone users in the world is expected to pass the five billion by 2019. Globally; internet coverage rate is 56, 8%. Despite of these facts people in emergency response need reaches Command and Control Centers (CCC) through voice call which causes time delays. These time delays could be dangerous in case of life threatening emergencies. So the Next Generation Emergency Services (NEXES) which was a Research and Innovation Action was granted by European Commission. NEXES aims to research, test and validate the promising integration of IP-based communication technologies and interoperability into the next generation emergency services, so that they attain increased effectiveness and performance. Empowered by smartphones with cameras, messaging and internet-based applications connecting to social media, citizens expect emergency services to use the same technologies. NEXES innovates the approach to the dynamics between emergency services and citizens, allowing (i) the use of total conversation capabilities in emergencies, including social media, to the benefit of citizens, including those with disability or special needs (ii) the exploitation of improved location information to rapidly and effectively identify and locate the caller and the incident site and (iii) the leverage of Internet-enabled connectivity to enhance interoperability and shared awareness among emergency services, to the benefit of a more secure society. Testing in a real life setting and giving feedbacks was a part of the project. Accessibility of every citizen to emergency services was one of the main goal of the project. We consider not only injured or disabled people are vulnerable for accessibility but also sometimes people can be transiently impaired for example after a dentistry operation. After the completion of NEXES Application we test it in a real life setting. Two cars placed like they are crashed and a virtual command prepared. Participants got roles like; virtual CCC staff, first responders, driver (the crashed victim who was also cannot speak due to dental surgery) and citizens etc. After the crash the driver was unconscious but his car sent automated call to virtual CCC. Few minutes later driver’s situation was changed he was conscious but he was unable to speak. He activated his NEXES application in his smart phone. At the same time his medical info and location info transferred to the virtual CCC. Dispatcher started and starts total conversation which enables real time audio and video call, text message features. Dispatchers understood the traffic collision and the symptoms/findings of the trapped victim and dispatched the suitable vehicles and equipment and gave first aid instructions to stop bleeding to the trapped victim. After the testing verbal feedbacks collected from the victim and first responders (and dispatchers) to improve the features of the NEXES Application. 1) First Responder Feedbacks: 1.1) Advantages; • NEXES Application improves situational awareness of first responders and dispatchers. • Dispatcher can see the situation easily and decide what to do which vehicle and team to dispatch to the scene. • Enables communication • Dispatchers can get more info from the scene in mass causality incidents • Reduces response time • Easy to give post-dispatch instructions • Easy to correct interventions if bystanders doing inadequate or wrong • First aid interventions start early in the scene • Quick and easy usage • It improves also first responders and callers’ /citizens’ communication and enables situational awareness for PSOs/first responders • Increases survival rate 1.2) Disadvantages; • When more video calls received more dispatchers may be necessary • When you want to show something so you must turn your telephone, there is no option to change the way of camera. • Dependant to internet • Dependant to technology (you need a smart phone) • The application must be downloaded before and citizens must be trained • Out of battery or low/poor internet or internet disconnection • If PSAP operator gets another video call both conversations can be heard • People who do not use smart telephone will be excluded • Network may cause problem 1.3) Suggestions; • The front or back cameras of the phone must be selected optionally • Video recording feature could be necessary • Photo recording feature could be necessary • Language options must be prepared • Calling from computers, laptops and/ or tablets must be achieved • Patient medical history must be seen in the PSAP’s screen • It must be tested in mass causality incident environment • May be another system can be improved apart from the internet • Public training has great importance • Needs media adds for dissemination 2) Citizen Feedbacks: 2.1) Advantages; • Real time video call, text message and voice call features are very helpful for people in need of help • Rapid response of first responders could be provided • Improved accessibility for deaf or blind people • Better communication between EROs and citizens • Provides interactive response to the people in need • Buying time until arriving of first responder teams (giving necessary medical instructions to the bystanders) • Providing text message feature • NEXES Mini App easy to use for people in panic 2.2) Disadvantages; • Training is necessary for citizens • Dependant to internet speed • Location information cannot be suitable for multi-layer buildings • When people pushes NEXES App closure key, the app closes immediately so it can happen accidentally, it is better to add an option like “are you sure to close NEXES App?” So people can choose “yes” or “no” options. • Only one camera can be used, using both cameras will be more helpful • More training is needed for elder people 2.3) Suggestions; • Automatic location information must be provided if the location status is closed on the phone • “Enter” button must be added for sending text massages because people cannot understand if the text message is sent or not • The text fond must be bigger • Interfaces could be developed in order to make the app easy to use • Information notes can be added • Instructive videos can be added • Educational videos must be prepared for esp citizens in order to enhance public training • Many people have smart televisions so if NEXES App can be adapted to smart tvs it will be easier to for elder people and children • More cooperation is necessary with telephone service providers.

**Conclusions:**

NEXES Application enhances post-crash response time and quality.

**Keywords:**

Post-crash response, Next generation, Emergency Services

